# PACS2 at Mitochondria‐Associated Membranes: A Central Regulator of Cardiovascular and Metabolic Diseases

**DOI:** 10.1111/jcmm.71336

**Published:** 2026-08-26

**Authors:** Baiyi Tang, Hui Wang, Jie Ouyang

**Affiliations:** ^1^ Department of Cardiology The Third Xiangya Hospital of Central South University Changsha Hunan China; ^2^ Intensive Care Unit Yueyang Central Hospital Yueyang Hunan China

**Keywords:** cardiovascular and metabolic diseases (CVMDs), mitochondria‐associated membranes (MAMs), PACS2

## Abstract

Cardiovascular and metabolic diseases (CVMDs), which are characterized by metabolic dysregulation and cardiovascular impairment, constitute a major global cause of mortality, thus highlighting the urgent need for novel therapeutic strategies. Phosphofurin acidic cluster sorting protein 2 (PACS2), a multifunctional adapter protein localized to mitochondria‐associated membranes (MAMs), regulates intracellular membrane trafficking and is pivotal for critical cellular processes, including apoptosis, autophagy and ferroptosis. Accumulating evidence implicates PACS2 dysregulation in the pathogenesis of diverse CVMDs, including metabolic disorders and cardiovascular conditions. This review examines the structural and functional basis of PACS2 and details its established and emerging roles in the pathogenesis of CVMDs. Collectively, these findings identify PACS2 as a crucial regulator of CVMD development and progression, underscoring its significant potential as both a diagnostic biomarker and a promising therapeutic target.

## 1 Introduction

Cardiovascular and metabolic diseases (CVMDs) represent the leading cause of death worldwide [1]; these diseases result from complex interactions among multiple pathological factors whose pathogenesis remains incompletely understood. Despite significant advancements in CVMD prevention and treatment over the past two decades, associated morbidity and mortality rates remain high [2, 3]. Consequently, it is imperative to identify novel pathogenic mechanisms and develop alternative therapeutic strategies for CVMDs.

Organelles, as fundamental cellular components, are critically involved in tissue metabolism and the pathogenesis of diverse diseases. Mitochondria‐associated membranes (MAMs), specialized lipid raft‐like domains formed at contact sites between the endoplasmic reticulum (ER) and mitochondria, comprise segments of both the ER and mitochondrial outer membranes [4]. Research in recent decades has identified MAMs as crucial hubs for regulating essential cellular processes, including Ca^2+^ signalling, lipid transport and metabolism, mitochondrial dynamics, inflammatory responses, apoptosis and autophagy [5, 6]. Increasing evidence further implicates MAM dysregulation in the development of CVMDs, although the precise underlying mechanisms require further elucidation [7].

The structural integrity and functional capacity of MAMs are governed by key regulatory proteins. Among these proteins, phosphofurin acidic cluster sorting protein 2 (PACS2) stands out as a pivotal MAM regulator. The PACS family was initially identified in lower metazoans, where a single PACS gene orchestrates membrane trafficking in invertebrates and lower chordates. Vertebrates, however, possess two paralogous genes, PACS1 and PACS2 [8]. PACS2, in particular, extends beyond its role in membrane trafficking to significantly influence critical cellular activities [8, 9].

Previous reviews have primarily summarized the biological functions of PACS2 as a key regulator of mitochondria‐associated membranes (MAMs). In recent years, however, the role of PACS2 has expanded far beyond organelle tethering and intracellular trafficking. Emerging evidence has implicated PACS2 in multiple cardiovascular and metabolic diseases through the regulation of apoptosis, autophagy, ferroptosis, lipid metabolism and cellular stress responses. More importantly, PACS2 appears to exert distinct, and sometimes opposing, effects depending on the cell type and pathological context. As research in this area continues to grow rapidly, an updated synthesis of current evidence is warranted. In this review, we summarize the emerging roles of PACS2 across cardiovascular and metabolic diseases, discuss the underlying molecular mechanisms and highlight its potential translational value as a diagnostic and therapeutic target.

## 2 Structure and Function of PACS2

### 2.1 Structure of PACS2

The PACS protein family was initially identified through genetic screens for modulators of furin protease trafficking within the secretory pathway [10, 11]. Mammals express two PACS paralogs: PACS1 and PACS2. The human *PACS2* gene is located on chromosome 14q32.33 and comprises 24 exons, generating at least 11 alternatively spliced transcripts. In comparison, the *PACS1* gene resides at chromosome 11q13.1–q13.2 and produces at least 12 alternative splice variants [8].

**FIGURE 1:**
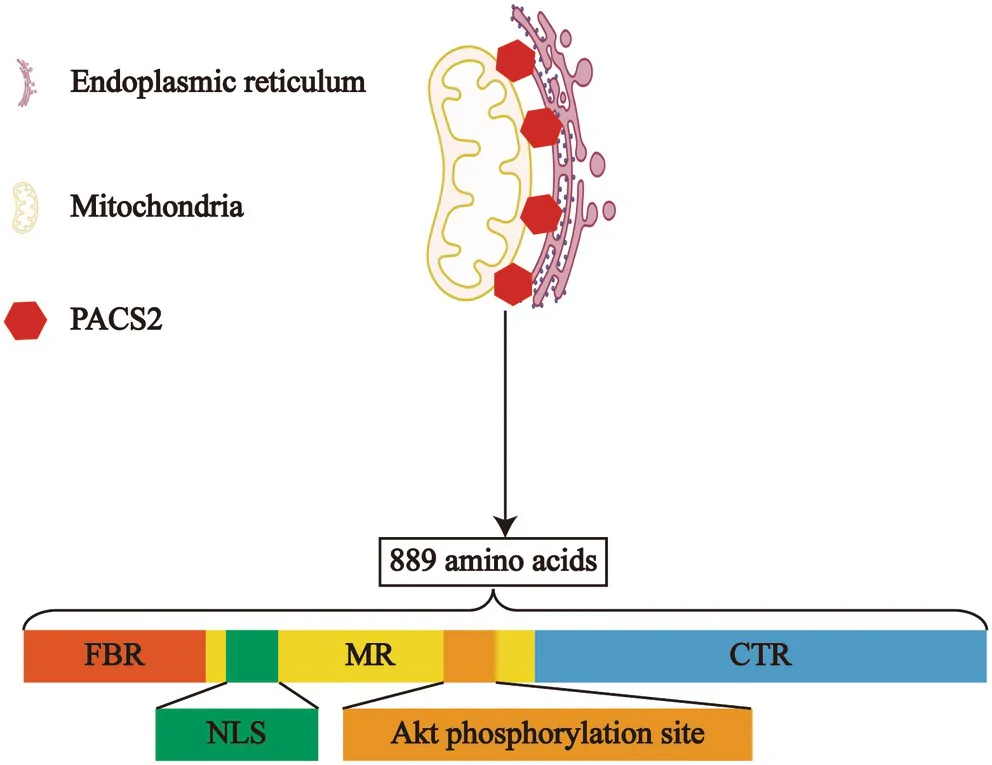
Schematic of the PACS2 structure in MAMs. CTR, C‐terminal region; FBR, furin‐binding region; MR, middle region; NLS, nuclear localization signal.

Structurally, both PACS proteins share a common domain organization, consisting of an N‐terminal furin‐binding region (FBR), a disordered middle region (MR) and a C‐terminal region (CTR) [8]. PACS2 is an 889‐amino acid protein, while PACS1 consists of 963 residues, with a 54% overall shared sequence identity. Notably, the FBR domains of PACS1 and PACS2 exhibit a high degree of conservation, with 81% sequence identity, and this domain is critical for cargo recognition and binding [9]. The MR of PACS2 harbours key functional motifs, including a nuclear localization signal (NLS) and an Akt phosphorylation site. Both motifs are essential for mediating PACS2 functions, such as facilitating ER–mitochondria communication and regulating apoptosis [9]. In contrast to the well‐characterized FBR and functionally significant MR, the specific role of the CTR remains unexplored (Figure 1).

### 2.2 Functions of PACS2

#### 2.2.1 Membrane Trafficking

As a key MAM‐resident protein, PACS2 plays an indispensable role in membrane trafficking and in mediating communication between organelles, the cytoplasm and membrane, and the cytoplasm and nucleus. For instance, PACS2 knockdown disrupts calnexin distribution within the ER, increasing its levels at the cell surface [12]. PACS2 is also essential for trafficking viral mitochondria‐localized inhibitor of apoptosis (vMIA) to the outer mitochondrial membrane (OMM). Superresolution imaging revealed that PACS2‐mediated membrane attachment or hydrophobic interactions alter the ability of vMIA to form nanoscale clusters at the OMM [13]. Furthermore, PACS2 recognizes the acidic cluster in the carboxy‐terminal domain of polycystin‐2 (a calcium‐permeable, nonselective cation channel). Upon binding, PACS2 facilitates polycystin‐2 transport between the ER, Golgi apparatus and plasma membrane, thereby regulating ion channel activity [14]. Dephosphorylation of PACS2 at Ser437 impairs its trafficking function and disrupts polycystin‐2 subcellular localization [15]. PACS2 also binds acidic residues in the carboxy‐terminal tail of the transmembrane ubiquitin ligase K5, which is required for K5‐mediated ubiquitination of cell surface CD31 and its subsequent lysosomal degradation [16]. Additionally, cytosolic PACS2 translocates into the nucleus and interacts with SIRT1 to inhibit SIRT1‐mediated p53 deacetylation [17]. PACS2 further prevents ADAM17 degradation and promotes its trafficking to the cell surface, thereby activating ADAM17. Consequently, PACS2 loss reduces ADAM17 surface levels and ADAM17‐dependent ErbB ligand shedding without significantly affecting related proteases [18]. Moreover, PACS2 interacts with transcription factor EB (TFEB), promoting TFEB nuclear translocation. Nuclear TFEB activates FAM134B, inducing ER autophagy to clear damaged ER components and maintain homeostasis [19]. Finally, Drosophila PACS2 colocalizes with GOLPH3 at the Golgi apparatus in primary spermatocytes, mediating vesicular trafficking, which is essential for furrow ingression during cytokinesis [20].

**FIGURE 2:**
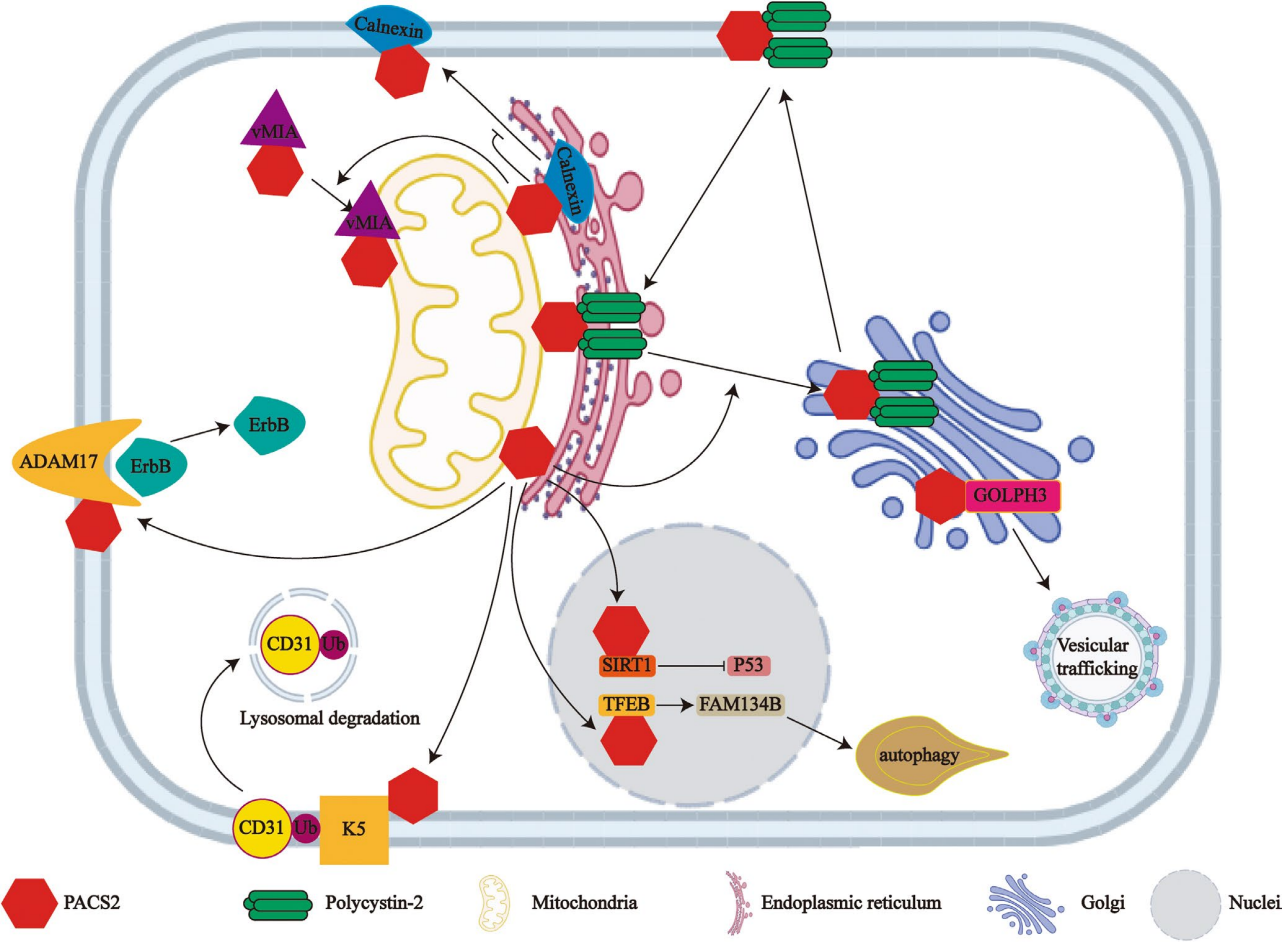
Schematic representation of the role of PACS2 in intracellular membrane trafficking.

These multifaceted roles establish PACS2 as a critical regulator of membrane dynamics, organelle communication and cellular homeostasis—processes highly relevant to CVMDs (Figure 2).

#### 2.2.2 Apoptosis

Apoptosis is a pivotal process in the pathogenesis of CVMDs. PACS2 plays a context‐dependent role in apoptotic regulation, functioning as either a pro‐ or antiapoptotic factor, with significant implications for disease mechanisms.

The pivotal role of PACS2 in apoptosis was first identified by Simmen et al. PACS2 binds to a dephosphorylated Bcl‐2‐interacting domain death agonist (Bid) and facilitates its transfer to mitochondria. Subsequent cleavage of Bid generates truncated Bid (tBid), triggering cytochrome c release and caspase‐3 activation, thereby ultimately leading to apoptosis [9]. Further studies revealed that PACS2 phosphorylation at Ser437 is a key regulatory node in apoptosis induced by TNF‐α‐related apoptosis‐inducing ligand (TRAIL). TRAIL triggers Ser437 dephosphorylation, activating the pro‐apoptotic function of PACS2. Conversely, Akt‐mediated phosphorylation of Ser437 enhances 14‐3‐3 binding to PACS2, inhibiting its apoptotic activity [15]. In Huh‐7 cells, TRAIL induces PACS2 to form immunoprecipitation complexes with Bim and Bax on lysosomal membranes. This complex promotes lysosomal membrane permeabilization (LMP) and apoptosis. Notably, PACS2 knockdown prevented Bim/Bax lysosomal recruitment, attenuated TRAIL‐induced LMP and enhanced cell survival [21]. Additionally, the E3 ligase cIAP1/2 blocks TRAIL‐induced apoptosis by ubiquitinating and downregulating PACS2 [22].

Accumulating evidence suggests that modulating PACS2 is a significant mechanism with respect to apoptosis. For instance, miRNA‐182 inhibits cardiomyocyte apoptosis in nonischemic heart failure by downregulating PACS2 expression [23]. Similarly, miRNA‐499 suppresses cardiomyocyte apoptosis by inhibiting PACS2 expression, consequently blocking Bid expression and mitochondrial translocation [24]. In oocytes from mice with high‐fat diet (HFD)‐induced obesity, PACS2 downregulation reduced mitochondrial Ca^2+^ levels, decreased apoptosis and promoted cytoplasmic maturation [25]. Conversely, a HFD upregulated PACS2 expression and increased MAM contacts in hepatocytes, promoting rapid ER‐to‐mitochondria Ca^2+^ transfer and apoptotic signalling [26]. Consistent with these findings, dietary nitrate supplementation inhibited the elevated PACS2 expression and apoptosis observed in the testes of rats with streptozotocin (STZ)‐induced diabetes [27, 28]. Furthermore, oxidized low‐density lipoprotein (Ox‐LDL) increased PACS2, MAMs and apoptosis in endothelial cells (ECs). PACS2 silencing significantly attenuated Ox‐LDL‐induced apoptosis, mitochondrial Ca^2+^ overload and reactive oxygen species (ROS) production [29]. Mechanistically, circ_0002194 promotes endothelial cell apoptosis after Ox‐LDL treatment partly by sponging miR‐637 to upregulate PACS2 expression [30]. Targeting PACS2 has also been reported to have therapeutic potential; oxyphyllanene B overcomes temozolomide resistance in glioblastoma partly by activating PACS2‐mediated apoptosis [31]. Moreover, PACS2 is upregulated during acute traumatic brain injury, and its silencing alleviates mitochondria‐derived oxidative stress and inhibits inflammation, ER stress and apoptosis [32].

Interestingly, several studies have reported antiapoptotic effects of PACS2. For example, Barroso‐González et al. demonstrated that PACS2 knockdown in mouse primary thymocytes attenuated Bcl‐xL induction, increased mitochondrial outer membrane permeability (MOMP), and accelerated apoptosis during DNA damage [33]. Biochemical analyses confirmed that PACS2 interacts with ataxia telangiectasia mutated (ATM) kinase to activate an antiapoptotic pathway upon DNA damage, driving NF‐κB activation and Bcl‐xL expression [33]. In idiopathic pulmonary fibrosis, PACS2 reduces alveolar epithelial cell apoptosis [34]. Similarly, Ox‐LDL treatment increased PACS2‐associated MAM contacts in vascular smooth muscle cells (VSMCs), and PACS2 knockdown increased apoptosis [35]. Additionally, PACS2 knockdown exacerbated renal cell apoptosis in diabetic nephropathy [36].

**FIGURE 3:**
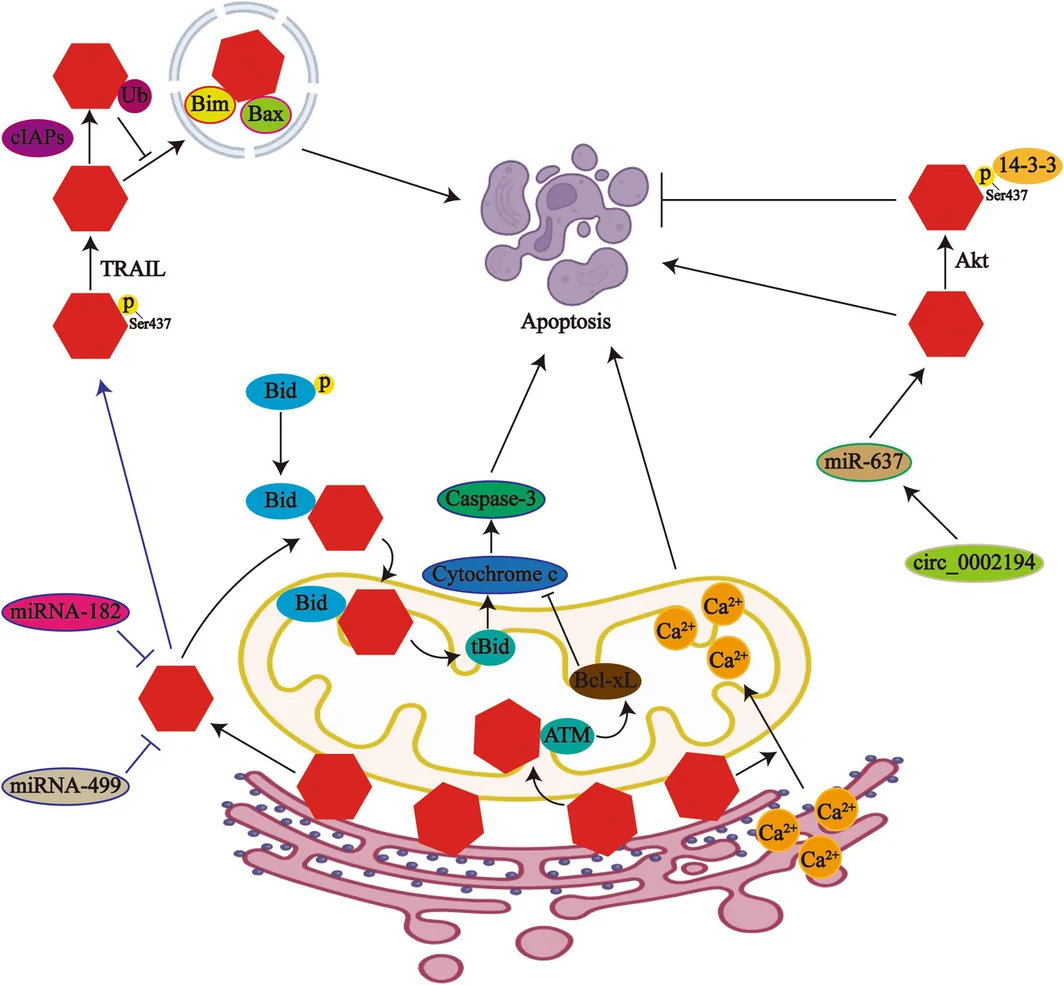
Schematic depicting the context‐dependent dual regulatory role of PACS2 in apoptosis.

Collectively, PACS2 plays a dichotomous role in apoptosis, acting as either a promoter or inhibitor of programmed cell death depending on the cellular context, stimuli and interacting partners. Its pro‐apoptotic function is primarily mediated through MAMs, facilitating the mitochondrial translocation of pro‐apoptotic proteins such as Bid and promoting MOMP and caspase activation. Conversely, PACS2 can exert antiapoptotic effects by maintaining MAM integrity, facilitating prosurvival signalling and regulating processes such as autophagy. Based on the evidence reviewed, the direction of PACS2‐mediated apoptotic regulation can be predicted by considering several key determinants, such as cell type, disease or physiological context, specific stimuli, post‐translational modifications, interacting partners, subcellular localization and MAM dynamics. This functional duality underscores the importance of tissue‐ and disease‐specific regulation of PACS2 in CVMDs (Figure 3).

#### 2.2.3 Autophagy

Autophagy is a conserved process that is essential for cellular homeostasis and involves the degradation and recycling of proteins and organelles. Autophagy plays a complex role in CVMDs, and PACS2 has been identified as a meaningful modulator of autophagic flux. While generally protective, dysregulation of autophagy machinery or excessive flux can lead to cell death [37]. Accumulating evidence implicates PACS2 as a key regulator of autophagy.

For instance, in diabetic nephropathy, PACS2 interacts with TFEB, promoting its nuclear translocation. Nuclear TFEB subsequently activates FAM134B expression, facilitating ER autophagy, reducing ER damage in renal tubular cells and ameliorating disease progression [19]. Furthermore, PACS2 enhances mitophagy by inhibiting the recruitment of dynamin‐related protein 1 (DRP1) to mitochondria, thus providing an additional protective mechanism in diabetic nephropathy [38]. Conversely, in Ox‐LDL‐treated VSMCs, PACS2 knockdown disrupts MAM contacts, impairing autophagosome formation and autophagy [35].

Collectively, these findings underscore the pivotal role of PACS2 in modulating autophagy across diverse pathological contexts. By regulating key processes such as ER autophagy and mitophagy and maintaining MAM integrity, PACS2 functions as a critical mediator of cellular homeostasis. Its involvement in autophagy‐related pathways highlights its potential as a therapeutic target for CVMDs characterized by autophagic dysfunction.

#### 2.2.4 Ferroptosis

Ferroptosis, an iron‐dependent, lipid peroxidation‐driven form of regulated cell death, plays a critical role in diverse pathologies. Recent studies have implicated mitochondrial dihydroorotate dehydrogenase (DHODH) as a key modulator of ferroptosis [39]. Given its localization at MAMs, PACS2 is positioned to influence ferroptosis pathways. Our recent research demonstrated that PACS2 inhibits DHODH via CPT1A, promoting ferroptosis in cardiomyocytes and contributing to the development of diabetic cardiomyopathy [40].

These findings highlight the emerging regulatory role of PACS2 in ferroptosis, particularly within CVMDs. The identification of PACS2 as a novel modulator of ferroptosis through DHODH inhibition provides crucial mechanistic insight and suggests promising therapeutic targets for diabetic cardiomyopathy and related disorders.

## 3 PACS2 in Metabolic Diseases

Metabolic diseases encompass a broad spectrum of disorders affecting multiple organs, including the liver, kidney, heart and reproductive system. Despite their diverse clinical manifestations, these conditions share common pathological features, particularly impaired energy metabolism, lipid accumulation, insulin resistance and mitochondrial dysfunction. Increasing evidence suggests that mitochondria‐associated membranes (MAMs) serve as a central hub integrating these metabolic signals.

As a key component of MAMs, PACS2 has been implicated in regulating metabolic homeostasis across different tissues. However, its functional consequences appear highly context dependent, varying according to cell type and metabolic demands. In the following sections, we summarize the role of PACS2 in major metabolic diseases, with a focus on its involvement in organ‐specific metabolic regulation and mitochondrial function.

### 3.1 PACS2 in Obesity and Associated Hepatic Steatosis

Obesity, a major risk factor for metabolic disorders, including insulin resistance, type 2 diabetes and nonalcoholic fatty liver disease (NAFLD), disrupts cellular organelle homeostasis and signalling pathways. Central to these disruptions is PACS2 dysregulation. This multifunctional protein emerges as a critical node in obesity‐associated pathologies, impacting both systemic metabolism and dysfunction in specific organs, particularly in the liver.

In the liver, the primary metabolic organ, obesity triggers pathological reorganization of MAMs. This reorganization causes mitochondrial calcium ([Ca^2+^]_m_) overload, impaired oxidative phosphorylation, and elevated oxidative stress. Critically, experimental induction of ER–mitochondrion tethering exacerbates metabolic dysfunction. Conversely, downregulating PACS2 restores mitochondrial oxidative capacity and improves glucose metabolism in obesity models [41]. These findings identify PACS2‐mediated MAM hyperconnectivity as a driver of hepatic mitochondrial dysfunction and systemic metabolic impairment in obesity.

In addition to facilitating organelle tethering, PACS2 regulates critical metabolic signalling in obesity. In the liver, PACS2 is integral to the insulin‐dependent diurnal inhibition of SIRT1, an NAD^+^‐dependent deacetylase that is central to energy homeostasis. Insulin signalling triggers a phosphorylation cascade on SIRT1 involving CK2 and GSK3, promoting its interaction with DBC1. This complex formation facilitates the PACS2‐dependent inhibition of nuclear SIRT1 enzymatic activity and its cytoplasmic translocation. Mechanistically, defects in this DBC1/PACS2‐controlled SIRT1 inhibitory pathway contribute to the development of obesity and NAFLD, implicating PACS2 in the dysregulation of fuel substrate switching [42].

The pathological role of PCAC2 in the obese liver is further highlighted by its contribution to increased susceptibility to hepatotoxic insults. Studies using the environmental toxin BDE‐209 have demonstrated that a HFD‐induced obese state significantly exacerbates liver injury. Mechanistically, a HFD increases PACS2 protein expression and expands the ER, increasing ER–mitochondria interactions. This enhanced MAM connectivity promotes excessive Ca^2+^ flux from the ER to mitochondria, activating both ER stress pathways and intrinsic mitochondrial apoptosis pathways and ultimately leading to severe hepatocyte apoptosis. Crucially, PACS2‐dependent MAM enrichment and Ca^2+^ dysregulation exacerbate BDE‐209 hepatotoxicity in the context of diet‐induced obesity [26].

The detrimental effects of obesity and PACS2 dysregulation are not confined to metabolic organs. Maternal obesity compromises oocyte quality, characterized by elevated MAM levels and increased expression of PACS2 and IP3Rs in oocytes. This aberrant MAM enrichment also drives [Ca^2+^]_m_ overload, promoting apoptosis and impairing cytoplasmic maturation—a critical process for developmental competence. Notably, PACS2 knockdown in obese oocytes effectively reduced MAM formation, normalized [Ca^2+^]_m_, suppressed apoptosis and rescued maturation defects. This finding identifies PACS2 as a central regulator of MAM‐driven cellular stress in reproductive tissues affected by obesity [25].

Collectively, these studies identify PACS2 as a pivotal pro‐obesity factor with multifaceted roles. It sustains excessive MAM tethering, leading to calcium dysregulation, mitochondrial dysfunction, oxidative stress and apoptosis across diverse cell types. Additionally, PACS2 regulates key metabolic signalling nodes, such as the DBC1/SIRT1 axis, contributing to hepatic insulin resistance and fuel metabolism dysregulation. Critically, PACS2 upregulation in obesity exacerbates the vulnerability of the liver to additional insults, such as environmental toxins. Consequently, PACS2 has emerged not only as a central mediator of obesity‐induced organelle stress, metabolic derangement and reproductive defects but also as a highly promising therapeutic target for mitigating a spectrum of obesity‐associated complications, including NAFLD and infertility. Collectively, these findings suggest that PACS2 may influence hepatic metabolic homeostasis by modulating MAM‐mediated lipid transfer and mitochondrial fatty acid oxidation, thus linking endoplasmic reticulum function with systemic energy balance.

### 3.2 PACS2 as a Key Regulator of Insulin Signalling

The direct involvement of PACS2 in insulin‐responsive pathways epitomizes the intricate relationship between organelle homeostasis and metabolic signalling. Emerging evidence positions PACS2 not only as a modulator of organelle dynamics but also as a critical transducer of insulin activity, primarily through its regulation of the metabolic sensor SIRT1.

SIRT1, an NAD^+^‐dependent deacetylase that is central to energy metabolism and insulin sensitivity, undergoes complex posttranslational regulation beyond cosubstrate availability. Crucially, its disordered N‐terminal region (NTR) functions as an insulin‐responsive sensor. This NTR contains an acidic cluster (AC) and a 3‐helix bundle (3HB) that regulate catalytic activity. Insulin signalling initiates a cascade in which the allosteric assistor DBC1 binds the SIRT1 NTR, removing a distal N‐terminal shield from the 3HB. This conformational change enables PACS2 to engage both the AC and the transiently exposed helix 3 of the 3HB. PACS2 binding disrupts the structural integrity of the 3HB, thereby profoundly inhibiting SIRT1 deacetylase activity [43]. This PACS2‐dependent inhibition represents a direct, insulin‐triggered mechanism for suppressing SIRT1 function, thus facilitating the metabolic shift towards glucose utilization.

Furthermore, studies on agents that mitigate mitochondrial dysfunction reinforce the link between organelle stress and insulin resistance. Alpha‐lipoic acid (ALA), which has been reported to improve insulin sensitivity, has protective effects against ER stress‐induced insulin resistance in hepatic cells. Although ALA does not directly alleviate ER stress signals, it rescues mitochondrial function by restoring oxidative phosphorylation (OXPHOS) complex expression, increasing ATP production and increasing mitochondrial β‐oxidation capacity. Critically, the ability of ALA to restore insulin sensitivity under conditions of ER stress directly correlates with improved mitochondrial performance [44]. This finding underscores that mitochondrial dysfunction—often downstream of PACS2‐mediated MAM dysregulation and Ca^2+^ overload—is a pivotal contributor to insulin resistance.

Collectively, these findings suggest that PACS2 is a master regulator that integrates insulin activity with cellular organelle homeostasis. Its dual role—inhibiting the key metabolic stabilizer SIRT1 and driving ER–mitochondria miscommunication—establishes PACS2 as a central factor in the development of insulin resistance. These observations indicate that PACS2 may indirectly regulate insulin signalling by affecting MAM integrity and downstream metabolic stress pathways, highlighting its role as an upstream modulator of cellular insulin sensitivity. Consequently, targeting the PACS2‐SIRT1 interaction represents a promising therapeutic strategy for insulin‐associated metabolic diseases. Although these findings support an important role for PACS2 in insulin signalling, the current evidence is largely derived from experimental models, and its clinical relevance in human metabolic disorders remains unclear.

### 3.3 Role of PACS2 in Renal Dysfunction

Renal tubular injury is a common pathological manifestation of metabolic disorders that can be triggered by environmental stressors. Recent studies have implicated dysregulation of mitochondria‐associated membranes (MAMs) in the development of renal injury, highlighting the importance of organelle communication in maintaining tubular homeostasis [45, 46]. As a key MAM scaffolding protein, PACS2 has been shown to participate in Cu‐induced nephrotoxicity by regulating ER–mitochondria interactions, mitochondrial function and cellular stress responses [45, 46]. Although these findings were obtained in a toxic injury model, they suggest that disruption of PACS2‐mediated MAM homeostasis may represent a common mechanism underlying renal metabolic dysfunction. Notably, similar mitochondrial and metabolic abnormalities are also observed under diabetic conditions, where hyperglycemia further amplifies MAM remodelling and cellular stress responses. These observations provide a mechanistic basis for understanding the role of PACS2 in diabetic nephropathy, as discussed in the following section.

### 3.4 Role of PACS2 in Diabetic Nephropathy (DN)

DN, a major microvascular complication, involves complex crosstalk among metabolic stress, mitochondrial dysfunction and aberrant lipid handling. Emerging evidence reveals a dichotomy in the role of PACS2 in the diabetic kidney, necessitating nuanced mechanistic dissection. While PACS2 is predominantly cytoprotective in the tubular epithelium, it has context‐dependent detrimental effects on other renal compartments.

The prevailing paradigm supports a renoprotective role for PACS2 in tubular epithelial cells. In diabetic mice, proximal tubule‐specific PACS2 knockout exacerbated tubulointerstitial injury, proteinuria and tubular lipid accumulation by dysregulating lipid metabolism. This manifested as increased SOAT1‐mediated cholesterol esterification, SREBP activation and impaired cholesterol efflux [47]. Human and murine DN kidney tissues exhibit reduced PACS2 expression, which is correlated with disrupted MAMs, mitochondrial fragmentation and decreased renal function [36, 38, 48, 49]. Mechanistically, proximal tubule‐specific PACS2 knockout increased MAM loss, ER stress, mitochondrial dysfunction and defective mitophagy. Conversely, tubule‐specific PACS2 overexpression restored MAM integrity, enhanced mitophagy via BECN1 relocalization and attenuated mitochondrial fission [36, 38]. Furthermore, PACS2 preserved ER homeostasis by promoting TFEB‐dependent ER autophagy and upregulating the ER autophagy receptor FAM134B, thereby mitigating ER stress [19]. Collectively, these data identify PACS2 as a guardian of tubular health, sustaining MAM‐dependent lipid flux, organelle quality control and ER proteostasis in DN.

Contrasting evidence suggests that PACS2 promotes renal endothelial dysfunction in DN. Diabetic mice exhibit elevated renal endothelial PACS2 expression along with microvascular hyperpermeability and glomerulosclerosis. Under high‐glucose/high‐fat conditions, PACS2 upregulation—driven by AKT activation—impaired endothelial fatty acid β‐oxidation (FAO). This process resulted in reduced ATP levels and NADPH/NADP^+^ ratios, and these effects were reversed by PACS2 silencing. Mechanistically, PACS2‐mediated FAO impairment promoted VE‐cadherin internalization and Smad2‐mediated fibrosis, positioning endothelial PACS2 as a driver of vascular injury in DN [50].

The opposing roles of PACS2 in the pathogenesis of DN likely stem from its cell type–specific functions. Within the renal tubular epithelium, PACS2 sustains MAM integrity, facilitating critical processes such as lipid transfer, calcium signalling and organelle crosstalk, which are essential for tubular energy metabolism and stress adaptation. Conversely, in glomerular endothelial cells, PACS2 appears to inhibit FAO, thereby exacerbating endothelial barrier dysfunction. These observations further suggest that the biological consequences of PACS2 may depend on the predominant function of MAMs in a given cell type. In metabolically active tubular epithelial cells, PACS2‐mediated MAM integrity appears to support mitochondrial quality control and lipid homeostasis, whereas in endothelial cells, excessive PACS2 activation may favour maladaptive metabolic remodelling. Therefore, the impact of PACS2 cannot be interpreted simply as protective or detrimental but should be considered within the specific cellular and pathological context. In diabetic nephropathy, PACS2 plays a more pronounced role in integrating hyperglycemia‐induced metabolic stress with mitochondrial dysfunction, thus contributing to tubular injury and glomerular endothelial impairment in a context‐dependent manner. Nevertheless, most mechanistic insights have been obtained from animal models and cultured cells. Whether these cell type‐specific functions of PACS2 are preserved in human diabetic nephropathy requires further clinical validation.

### 3.5 Role of PACS2 in Diabetic Cardiomyopathy (DCM)

DCM, a severe complication that is characterized by diabetes‐induced structural and functional cardiac abnormalities in the absence of hypertension or coronary artery disease, presents significant clinical challenges because of the scarcity of early diagnostic biomarkers and effective therapeutic targets. Emerging evidence has established PACS2 as a central regulator of the pathogenesis of DCM, orchestrating disease progression through its dual functions in modulating MAM integrity and ferroptosis.

Under hyperglycemic conditions, PACS2 is significantly upregulated in cardiomyocytes. This upregulation drives aberrant MAM formation, impairing mitochondrial biogenesis, fusion and OXPHOS. The resulting mitochondrial dysfunction activates the SMAC–HTRA2–ARTS–XIAP apoptosis cascade, directly contributing to cardiomyocyte loss and DCM progression. Notably, pharmacological inhibition of PACS2 by ferulic acid ameliorates DCM by normalizing PACS2‐mediated MAM alterations [51].

Furthermore, PACS2 critically regulates ferroptosis in DCM. PACS2 silencing markedly suppresses ferroptosis by downregulating the expression of the fatty acid transporter CPT1A and upregulating the expression of the mitochondrial enzyme DHODH. This coordinated action reduces iron accumulation, lipid peroxidation and ROS generation, delineating a novel PACS2/CPT1A/DHODH signalling axis that drives ferroptosis in DCM [40].

Collectively, these findings identify PACS2 as a multifunctional scaffold protein pivotal to DCM development. PACS2 integrates MAM‐driven mitochondrial apoptosis and ferroptotic cell death through distinct downstream effectors, thus identifying PACS2 as a promising biomarker and therapeutic target for early DCM intervention. In diabetic cardiomyopathy, PACS2‐mediated MAM remodelling influences cardiomyocyte metabolic flexibility and mitochondrial homeostasis, suggesting that its functional outcome depends on the balance between energy demand and stress adaptation in the diabetic heart.

### 3.6 Role of PACS2 in Diabetic Testicular Complications

PACS2 has also been implicated in diabetic complications beyond the cardiovascular system. In STZ‐induced diabetic rats, increased PACS2 expression has been associated with enhanced germ cell apoptosis and testicular structural damage, suggesting that PACS2 participates in diabetes‐induced reproductive dysfunction [27, 28]. Mechanistically, PACS2 upregulation occurs together with reduced NO bioavailability and altered expression of apoptosis‐related regulators, supporting its involvement in metabolic stress‐induced cell death. Administration of sodium nitrate partially reversed these pathological changes while reducing PACS2 expression, further implicating PACS2 in this process [27, 28]. Although evidence remains limited, these findings extend the role of PACS2 beyond classical metabolic organs and further support its participation in mitochondrial stress responses under diabetic conditions. Given the primary focus of this review, this section is intended to provide mechanistic context, whereas the following sections discuss the more extensively investigated roles of PACS2 in diabetic nephropathy and diabetic cardiomyopathy.

## 4 PACS2 in Cardiovascular Diseases

### 4.1 Role of PACS2 in Atherosclerosis

Emerging evidence has established PACS2 as a critical regulator of EC, VSMC and macrophage dysfunction in atherogenesis. Ox‐LDL, a key atherogenic stimulus, upregulates PACS2 expression in vascular ECs, thereby triggering apoptosis through PACS2‐mediated regulation of MAMs. Mechanistically, Ox‐LDL‐induced PACS2 expression promotes MAM formation, enhances ER–mitochondria contact and facilitates pathological Ca^2+^ transfer. This cascade leads to mitochondrial membrane potential dissipation, ROS generation and cytochrome c release, ultimately culminating in EC apoptosis—a pivotal event in plaque initiation [29].

PACS2 expression is further modulated by noncoding RNAs. Specifically, circ_0002194 upregulates PACS2 expression by sponging miR‐637, thereby promoting endothelial dysfunction and contributing to atherosclerosis progression. The circ_0002194/miR‐637/PACS2 axis thus represents a novel regulatory mechanism in this disease [30].

In addition to its role in endothelial injury, PACS2 plays a crucial role in the process of determining VSMC fate. Depletion of PACS2 disrupts MAM integrity, impairs mitophagy and exacerbates VSMC apoptosis—processes that are central to plaque vulnerability and medial degeneration [35].

Furthermore, our recent studies have demonstrated that elevated PACS2 expression in macrophages promotes lipid uptake and accelerates atherosclerotic progression via the ‘ROS–PPARγ–CD36’ signalling axis. Importantly, we identified a novel self‐amplifying positive feedback loop between PACS2 and this pathway, thereby providing new insights into the sustained inflammatory response in atherosclerosis [52]. We also found that FTO reduces the expression of PACS2 by decreasing the m^6^A modification of PACS2, thereby improving the foamification of macrophages [53].

Collectively, these findings indicate that PACS2 exerts highly cell type‐specific effects during atherogenesis. While increased PACS2 activity promotes endothelial injury and macrophage foam cell formation, it is also required for maintaining mitophagy and survival in VSMCs. This apparent functional divergence likely reflects the distinct cellular demands for MAM signalling in different vascular cell populations. Consequently, therapeutic strategies targeting PACS2 in atherosclerosis may require precise cell‐specific modulation rather than global inhibition or activation. Despite the growing body of evidence, current studies have primarily focused on individual vascular cell types in experimental settings. Future investigations integrating multicellular interactions and clinical samples will be important for clarifying the overall contribution of PACS2 to plaque development and stability.

### 4.2 Role of PACS2 in Myocardial Injury

Myocardial injury, characterized by the loss of functional cardiomyocytes and disruption of cardiac tissue integrity, represents a pivotal pathological process underlying the progression of numerous cardiovascular diseases (CVDs). Emerging evidence also implicates PACS2 as a significant contributor to myocardial injury.

PACS2 acts as a direct target of the cardioprotective microRNA miR‐499. In neonatal rat cardiomyocytes under H_2_O_2_‐induced oxidative stress, miR‐499 inhibits PACS2, thereby attenuating cardiomyocyte apoptosis [24].

In pressure‐overloaded hearts, PACS2 contributes to pathological remodelling through mitochondrial oxidative stress. Renal denervation (RDN) ameliorates cardiac hypertrophy and fibrosis in rats with hypertensive heart failure (HF) by downregulating PACS2 and BACH1 expression. Mechanistically, norepinephrine (NE) stimulation upregulates PACS2 expression via the TGF‐β1/SMAD/SP1 pathway, thereby exacerbating mitochondrial ROS accumulation, depleting antioxidant enzymes and impairing mitochondrial function. PACS2 knockdown rescues NE‐induced hypertrophy and mitochondrial dysfunction, thus confirming its role as a driver of oxidative damage [54].

Conversely, PACS2 performs protective functions under chronic hypobaric hypoxia (HH). HH downregulates cardiac PACS2, thereby disrupting MAM integrity and ER‐mitochondria Ca^2+^ flux. This impairment suppresses mitophagy and compromises aerobic metabolism, thus leading to right ventricular dysfunction. PACS2 overexpression or cardiac‐specific knock‐in restores MAM‐mediated Ca^2+^ signalling, mitophagy and mitochondrial energy metabolism, thereby preserving cardiac function. These findings underscore the indispensable role of PACS2 in adaptive mitochondrial responses to hypoxia [55].

Collectively, the current evidence reveals a context‐dependent duality regarding the role of PACS2 in myocardial injury. PACS2 functions as a pro‐injury mediator under oxidative and hypertensive stress, promoting cardiomyocyte apoptosis and exacerbating mitochondrial oxidative stress and dysfunction, thereby contributing to hypertrophy and fibrosis. In contrast, under chronic hypobaric hypoxia, PACS2 acts as an essential protector by safeguarding MAM integrity and facilitating ER‐mitochondria Ca^2+^ flux, mitophagy and aerobic energy metabolism to preserve right ventricular function. This functional dichotomy highlights the crucial influence of the specific pathological milieu on PACS2 activity. These findings suggest that the pathological outcome of PACS2 activation may depend on whether MAM remodelling primarily supports adaptive mitochondrial maintenance or instead drives excessive oxidative stress and apoptotic signalling. Future research should explore the therapeutic potential of modulating PACS2 activity precisely to treat cardiovascular diseases.

## 5 Perspectives

Recent studies have considerably expanded our understanding of PACS2 in cardiovascular and metabolic diseases. However, an important theme emerging from current evidence is that the role of PACS2 appears to be highly context dependent. In different disease settings and cell types, PACS2 can exert distinct and sometimes opposing biological effects. For example, PACS2 promotes endothelial dysfunction and macrophage foam cell formation during atherosclerosis, whereas it is required for maintaining mitophagy and cellular survival in vascular smooth muscle cells. Similarly, PACS2 protects renal tubular epithelial cells by preserving mitochondrial homeostasis but contributes to metabolic dysfunction in glomerular endothelial cells. These observations suggest that PACS2 should not be viewed simply as a protective or pathogenic factor. Rather, it is a central regulator of mitochondria‐associated membranes (MAMs), and PACS2 influences a wide range of downstream processes, including calcium signalling, lipid metabolism, mitochondrial quality control, oxidative stress, apoptosis, autophagy and ferroptosis. Consequently, the ultimate pathological outcome of PACS2 activation is likely determined by the specific cellular context and disease microenvironment.

The critical role of PACS2 in CVMDs is increasingly recognized, but its potential as a therapeutic target or diagnostic biomarker remains underexplored. While no clinical‐stage drugs directly targeting PACS2 currently exist, the role of PACS2 as a pivotal regulatory node offers unique opportunities for therapeutic innovation. Future research should prioritize the task of identifying the tissue‐specific interactome and signalling networks of PACS2 in pathological contexts (e.g., in the heart, liver, kidney and vasculature). Such mechanistic insights are essential for efforts to design highly selective interventions.

Given the established role of PACS2 in maintaining MAM integrity—a process frequently dysregulated in CVMDs [5]—indirect targeting strategies warrant exploration. Small‐molecule modulators that stabilize MAM function could mitigate mitochondrial calcium dyshomeostasis, metabolic stress and cell death, thereby conferring multiorgan protection [56, 57].

Concurrently, translational validation of the clinical utility of PACS2 is imperative. Systematic profiling of circulating PACS2 isoforms (e.g., serum protein levels and posttranslational modifications) in longitudinal cohorts may reveal correlations with disease onset, progression or therapeutic response. Such noninvasive biomarkers could enable early‐risk stratification and subtype classification [58]. Additionally, tissue‐restricted nucleic acid therapies (e.g., siRNA delivered via organotropic vectors [59]) represent a promising—albeit safety‐intensive—paradigm for spatially precise PACS2 modulation in target organs (e.g., the liver and heart).

Although PACS2 has emerged as a promising biomarker and therapeutic target in cardiovascular and metabolic diseases, its clinical translation remains at an early stage. First, most current evidence is derived from experimental models, whereas validation in large clinical cohorts is still lacking. It therefore remains unclear whether alterations in PACS2 expression or activity consistently correlate with disease onset, progression, or prognosis across different patient populations. Future translational research should determine whether PACS2 can serve as a reliable biomarker or therapeutic target in well‐characterized patient cohorts. Second, the context‐dependent functions of PACS2 present an important challenge for therapeutic intervention. As discussed above, PACS2 may exert distinct and even opposing effects depending on the affected tissue, cell type and disease stage. Consequently, systemic modulation of PACS2 could lead to unintended effects in non‐target tissues. Furthermore, PACS2 functions as a central organizer of mitochondria‐associated membranes (MAMs), where it coordinates multiple physiological processes essential for cellular homeostasis. Therapeutic strategies targeting PACS2 should therefore preserve normal MAM function while selectively correcting disease‐associated signalling abnormalities. Addressing these challenges will be critical for translating the growing mechanistic insights into clinically applicable diagnostic and therapeutic strategies.

Ultimately, converging mechanistic studies (leveraging advanced gene editing and imaging technologies) with clinical resources (e.g., biobanks and genetic databases) through interdisciplinary collaboration will be pivotal in transitioning PACS2 from a mechanistic nexus to a therapeutically actionable target. An additional consideration for the clinical translation of PACS2 research is the potential impact of interspecies differences in mitochondrial biology. Although animal models have provided essential insights into the role of PACS2 in regulating MAM integrity, mitochondrial metabolism, and cellular stress responses, mitochondrial structure, bioenergetic capacity, and adaptive responses can vary substantially between experimental animals and humans. These differences may influence the interpretation of preclinical findings and the effectiveness of PACS2‐targeted interventions in clinical settings [60]. Therefore, future studies should incorporate human‐derived models, patient samples and comparative analyses across species to better define the translational relevance of PACS2‐mediated mechanisms in cardiovascular and metabolic diseases. Importantly, most of the current evidence regarding PACS2 in cardiovascular and metabolic diseases is derived from in vitro experiments and animal models. Human studies remain scarce, and the available clinical evidence is insufficient to establish PACS2 as a reliable biomarker or therapeutic target. Therefore, future investigations should prioritize validation in well‐characterized patient cohorts and clinical specimens to facilitate the translation of experimental findings into clinical practice.

## 6 Conclusion

PACS2, a pivotal regulator anchored at MAMs, orchestrates CVMDs through its intracellular transport functions and diverse cellular regulatory mechanisms. This multifaceted involvement positions PACS2 as a promising therapeutic target for CVMDs, although clinical validation remains an urgent need.

This review has focused on the direct mechanistic contributions of PACS2 to the pathogenesis of CVMDs. Critically, the role of PACS2 is context dependent, manifesting as detrimental or protective effects contingent upon the cell type, cellular milieu and specific pathological environment (Table 1). This functional duality highlights the complexity of targeting PACS2 but simultaneously reveals a wealth of potential molecular intervention points within the PACS2 signalling network. Moving from this conceptual approach to actionable therapeutic strategies requires a focus on specific, tractable targets within the PACS2 system; this includes modulating specific protein–protein interactions, such as disrupting the binding of PACS2 to pro‐apoptotic partners like Bid or stabilizing its association with survival regulators, targeting functional domains critical for its recruitment or activity at MAMs, exploiting upstream regulators such as specific miRNAs or kinases like Akt that control PACS2 expression, phosphorylation or degradation, and leveraging its context‐dependent expression or function to achieve tissue‐ or disease‐stage‐specific effects. Exploiting this network holds significant therapeutic potential for diverse CVMDs, including NAFLD, diabetic nephropathy, diabetic cardiomyopathy, atherosclerosis and heart failure.

**TABLE 1:** Role of PACS2 in CVMDs.

Disease	Major cell type	PACS2 expression	Major mechanism	Biological consequence	Overall role
Obesity/NAFLD	Hepatocyte	Upregulated	[Ca^2+^]_m_ overload	Hepatic steatosis	Pathogenic
Insulin resistance	Insulin‐responsive cells	Upregulated	Inhibiting SIRT1, driving ER–mitochondria miscommunication	Insulin resistance	Pathogenic
Diabetic nephropathy	Tubular cells	Downregulated	Lipid metabolism	Tubulointerstitial injury	Protective
Diabetic nephropathy	Glomerular endothelial cells	Upregulated	MAM alterations	Glomerular fibrosis	Pathogenic
Diabetic cardiomyopathy	Cardiomyocytes	Upregulated	Ferroptosis, mitochondrial dysfunction	Cardiac remodelling	Pathogenic
Atherosclerosis	ECs	Upregulated	ER stress, apoptosis	Plaque progression	Pathogenic
Atherosclerosis	Macrophages	Upregulated	Lipid uptake	Foam cell formation	Pathogenic
Atherosclerosis	VSMCs	Downregulated	Mitophagy	Plaque instability	Protective
Myocardial injury	Cardiomyocytes	Upregulated	Mitochondrial quality control	Cardiac remodelling	Pathogenic

However, therapeutic strategies targeting PACS2 require careful design. Achieving selectivity, minimizing off‐target effects and ideally employing localized delivery systems are crucial for mitigating adverse effects on other organs and systems. The development of modalities such as cell‐specific nanocarriers or ligand‐conjugated therapeutics could help direct interventions to affected targets such as the vascular endothelium, hepatocytes or cardiomyocytes, thereby enhancing efficacy while sparing organs in which PACS2 may play protective roles.

While research on PACS2 in CVMDs is increasing, clinical trials—particularly human studies—remain relatively scarce. Consequently, robust clinical data are essential to conclusively demonstrate the efficacy of PACS2‐based interventions, thus laying a foundation for their future application in the context of CVMD management.

## Author Contributions

**Baiyi Tang:** software, writing – original draft. **Jie Ouyang:** conceptualization, writing – review and editing. **Hui Wang:** software.

## Funding

The authors have nothing to report.

## Conflicts of Interest

The authors declare no conflicts of interest.

## Data Availability

The authors have nothing to report.
